# Risk Factors for Metachronous Isolated Peritoneal Metastasis after Preoperative Chemotherapy and Potentially Curative Gastric Cancer Resection: Results from the CRITICS Trial

**DOI:** 10.3390/cancers13184626

**Published:** 2021-09-15

**Authors:** Irene A. Caspers, Karolina Sikorska, Astrid E. Slagter, Romy M. van Amelsfoort, Elma Meershoek-Klein Kranenbarg, Cornelis J. H. van de Velde, Pehr Lind, Marianne Nordsmark, Edwin P. M. Jansen, Marcel Verheij, Johanna W. van Sandick, Annemieke Cats, Nicole C. T. van Grieken

**Affiliations:** 1 Department of Gastrointestinal Oncology, Netherlands Cancer Institute, 1066 CX Amsterdam, The Netherlands; i.caspers@nki.nl (I.A.C.); a.cats@nki.nl (A.C.); 2 Department of Pathology, Amsterdam UMC, Vrije Universiteit Amsterdam, Cancer Center Amsterdam, 1081 HV Amsterdam, The Netherlands; 3 Department of Biometrics, Netherlands Cancer Institute, 1066 CX Amsterdam, The Netherlands; k.sikorska@nki.nl; 4 Department of Radiation Oncology, Netherlands Cancer Institute, 1066 CX Amsterdam, The Netherlands; a.slagter@nki.nl (A.E.S.); r.v.amelsfoort@nki.nl (R.M.v.A.); epm.jansen@nki.nl (E.P.M.J.); Marcel.Verheij@radboudumc.nl (M.V.); 5 Department of Biometrics, Leiden University Medical Center, 2333 ZA Leiden, The Netherlands; w.m.meershoek-klein_kranenbarg@lumc.nl; 6 Department of Surgery, Leiden University Medical Center, 2333 ZA Leiden, The Netherlands; c.j.h.van_de_velde@lumc.nl; 7 Department of Oncology, Stockholm Söder Hospital, 118 83 Stockholm, Sweden; pehr.lind@sll.se; 8 Department of Oncology and Pathology, Karolinska Institute, 171 77 Stockholm, Sweden; 9 Department of Medical Oncology, Aarhus University, 8200 Aarhus, Denmark; marianne.nordsmark@rm.dk; 10 Department of Radiation Oncology, Radboud University Medical Center, 6525 GA Nijmegen, The Netherlands; 11 Department of Surgery, Netherlands Cancer Institute, 1066 CX Amsterdam, The Netherlands; j.v.sandick@nki.nl

**Keywords:** gastric cancer, peritoneal metastasis, multimodality treatment, metachronous, risk factors

## Abstract

**Simple Summary:**

Around 20% of gastric cancer patients develop peritoneal metastasis after preoperative chemotherapy and curative surgery. Patients with peritoneal metastasis as a single site of metastasis may potentially benefit from prophylactic strategies. In this post-hoc analysis of the international phase III CRITICS trial, we aim to identify factors that can distinguish patients at high risk for developing peritoneal metastasis as a single site. Diffuse or mixed histological subtype, tumors with serosal involvement (ypT4) and advanced lymph node stage (ypN3 or a tumor positive lymph node ratio >20%) were independent risk factors for isolated peritoneal metastasis after preoperative chemotherapy and curative surgery. The combination of these risk factors identifies a subgroup that may benefit from treatment strategies that aim to prevent peritoneal metastasis.

**Abstract:**

Gastric cancer (GC) patients at high risk of developing peritoneal metastasis (PM) as a single site of metastasis after curative treatment may be candidates for adjuvant prophylactic strategies. Here we investigated risk factors for metachronous isolated PM in patients who were treated in the CRITICS trial (NCT00407186). Univariable and multivariable analyses on both metachronous isolated PM and ‘other events’, i.e., (concurrent) distant metastasis, locoregional recurrence or death, were performed using a competing risk model and summarized by cumulative incidences. Isolated PM occurred in 64 of the 606 (11%) included patients. Diffuse or mixed histological subtype, ypT4 tumor stage and LN^high^ (ypN3 lymph node stage or a lymph node ratio >20%) were independent risk factors for isolated PM in both univariable and multivariable analyses. Likewise, LN^high^ was an independent risk factor for ‘other events’. Patients with tumors who were positive for all three independent risk factors had the highest two-year cumulative incidence of 43% for isolated PM development. In conclusion, diffuse or mixed histological subtype, ypT4 and LN^high^ were identified as independent risk factors for isolated PM in patients treated with preoperative chemotherapy followed by surgical resection. The combination of these factors may identify a subgroup that may benefit from PM-preventing treatment strategies.

## 1. Introduction

Gastric cancer is the third most common cause of cancer-related deaths worldwide [1]. Survival after potentially curative surgery remains low, frequently due to tumor recurrence in the peritoneal cavity. Several multimodality treatment regimens have been introduced over the last decades to improve survival and reduce gastric cancer recurrence rates. Perioperative chemotherapy is currently the standard of care throughout many parts of Europe and the United States, and has improved the five-year overall survival rates for resectable gastric cancer by up to 45% [2,3]. Nevertheless, disease recurrence is frequent. Despite the administration of preoperative chemotherapy, peritoneal metastasis incidences have not decreased over the last decades [4]. Peritoneal metastasis (PM) is accountable for 30–50% of metastatic spread after potentially curative resection [4,5,6]. In The Netherlands, a significant part of recurrences (23%) is caused by metachronous isolated PM [7].

Patients with PM have an extremely poor prognosis, with a median survival of 3 to 5 months [8,9]. Palliative chemotherapy probably gives only modest survival benefits [10]. Therefore, both normothermic and hyperthermic intraperitoneal chemotherapy (HIPEC) have been introduced in order to potentially prolong survival after PM diagnosis. Although initially promising results were reported, HIPEC treatment may only be meaningful in highly selected patients with, for example, very limited peritoneal disease [11,12,13,14]. The currently ongoing PERISCOPE-II trial investigates the effect of cytoreductive surgery (CRS) and HIPEC after preoperative chemotherapy compared to palliative chemotherapy in gastric cancer patients with tumor positive cytology and/or limited peritoneal metastasis [15]. Hypothesizing that, specifically, patients with limited peritoneal disease seem to benefit from HIPEC, prophylactic HIPEC treatment has been suggested to potentially prevent or postpone peritoneal metastasis [16,17]. However, HIPEC is associated with considerable morbidity [18] and, preferably, should be restricted to a select group of patients with a very high risk for developing isolated PM. An accurate prediction model to identify these patients at high risk for metachronous isolated PM that might benefit from prophylactic treatment is, therefore, needed.

Multiple studies have shown various clinicopathological characteristics to be associated with PM in gastric cancer. Both younger age, non-cardia tumor localization, diffuse histological subtype, pT4 tumor stage and the presence of lymph node metastases have been identified as independent risk factors for PM [4,6,19,20,21,22]. Many of these studies, however, reported factors associated with synchronous PM [20,21,22]. Furthermore, the few studies that reported risk factors for metachronous peritoneal metastasis were performed before the introduction of perioperative chemotherapy in 2006 [3]. It remains unknown to what extent preoperative chemotherapy may have prevented the development of peritoneal metastasis [4,19,22]. To date, studies evaluating risk factors for metachronous PM as a single site of metastasis after both preoperative chemotherapy and potentially curative resection are sparse. As a result, the contribution of the established risk factors to the risk of metachronous isolated peritoneal metastasis in gastric cancer treated with preoperative chemotherapy remains unclear.

In this post-hoc analysis, risk factors for the development of peritoneal metastasis as a single site of metastasis after potentially curative resection in patients treated with preoperative chemotherapy as part of the CRITICS trial treatment regimen were investigated.

## 2. Materials and Methods

### 2.1. Patient Selection

Patients were selected from the international CRITICS trial (NCT00407186). The outline of the CRITICS trial has been previously reported [23]. In brief, 788 patients with stage Ib-IVa (AJCC 6th edition) adenocarcinoma of the stomach were randomly assigned to receive neo-adjuvant chemotherapy and D2 surgery, followed by either adjuvant chemotherapy or chemoradiotherapy. (Neo-) adjuvant chemotherapy consisted of three cycles of epirubicin, cisplatin or oxaliplatin and capecitabine. Chemoradiotherapy consisted of 45 Gy in 25 fractions in combination with cisplatin and capecitabine. No survival differences were found in the intention-to-treat analysis [24].

For the current analysis, revision of both surgery and pathology reports were carried out. All surgery reports had previously been reviewed by two expert gastrointestinal surgeons [25]. All patients who underwent a potentially curative gastric cancer resection, irrespective of the start of postoperative treatment, were selected. Post-operative deaths, defined as death within 30 days after surgery, were excluded. If no signs of peritoneal disease were described after histopathological examination of the resection specimen, patients were included in the current analysis. Tumors found to be of neuro-endocrine origin after histopathological examination of the resection specimen were excluded. Results of both treatment arms were combined in our study, since no differences in survival were found in the intention-to-treat analysis of the CRITICS trial [24].

### 2.2. Follow Up in the CRITICS Trial

Patients were followed up every month during the first 3 months after post-operative treatment, and every 3 months in the remainder of the first year. Thereafter, follow-up visits were every 6 months until 5 years of follow-ups had occurred. Follow-ups included computed tomography every 6 months, or earlier if deemed necessary by the treating physician. All first events were recorded by the treating physicians and included locoregional, distant and peritoneal metastasis or death from any cause.

### 2.3. Pathological Characteristics

Pathological characteristics, i.e., tumor stage, lymph node stage, lymphatic invasion, vascular invasion and histological subtype according to Lauren [26], were retrospectively curated from original surgery and pathology reports and updated to the 8th edition of the AJCC TNM classification [27]. Based on risk factors reported in the literature, the following groups were defined: intestinal type GC versus diffuse/mixed type GC and ypT0-ypT3 versus ypT4. In order to include all patients with advanced lymph node metastases, irrespective of the number of resected lymph nodes, patients were grouped as LN^low^ or LN^high^ based on both the ypN lymph node stage (ypN3 according to AJCC 8th edition) and lymph node ratio (number of tumor positive lymph nodes divided by the total number of lymph nodes harvested). LN^high^ was defined as ypN3 lymph node stage and/or a lymph node ratio above 20%.

### 2.4. Outcome

The site of tumor recurrence was categorized as either locoregional, distant, peritoneal or at multiple sites. Events diagnosed within 30 days from each other were classified as recurrence at multiple sites. Isolated peritoneal metastasis was defined as the presence of tumor tissue on the peritoneum, greater or lesser omentum, transverse mesocolon, diaphragm and/or ascites, without signs of distant metastases. As previously reported, distant metastasis was defined as tumor tissue in other organs such as the liver, colon, lungs, pleura, gallbladder, duodenum, brain, bone, distant lymph nodes (stations 14–16) or ovaries [28]. Concurrent metastases on the peritoneum and ovaries were classified as isolated peritoneal metastasis in this analysis. Concurrent peritoneal metastasis and locoregional recurrence were classified as isolated peritoneal metastasis. Peritoneal metastasis in combination with a distant metastasis, only distant metastasis, only local recurrence or death without any recurrence reported were defined as ‘other events’ in this analysis. Time to peritoneal metastasis was defined as the time from potentially curative surgery until detection of peritoneal metastasis.

### 2.5. Statistical Analyses

Cumulative incidences of events were estimated using the competing risk methodology. We distinguished between metachronous isolated peritoneal metastasis and ‘other events’. Univariable and multivariable regression models were fitted for subdistributions of a competing risk using the Fine and Gray method. Risk factors significant in the univariable analysis at *p* ≤ 0.05 were further investigated in a multivariable analysis. Unknown histology was considered to be of diffuse or mixed type. Unknown lymphatic and vascular invasion was considered to be positive. In a sensitivity analysis, we also considered unknown histology to be of intestinal type, and lymphatic and vascular invasion to be negative, which led to virtually no changes in the estimated parameters. All analyses were performed using IBM SPSS statistical software version 25 (IBM Corp., Armonk, NY, USA) and R version 3.5.1 (R Core Team, Vienna, Austria).

## 3. Results

**Figure 1:**
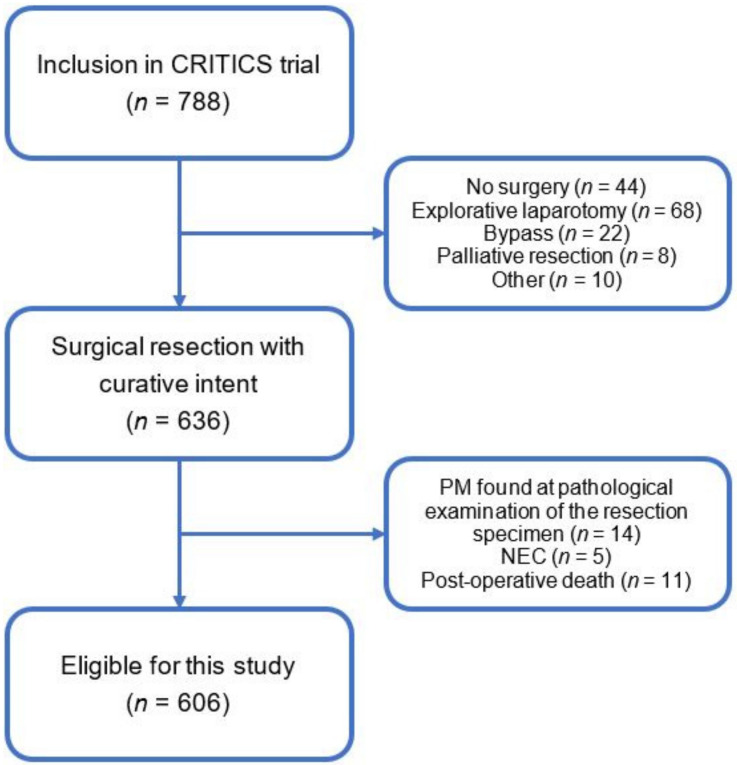
Flowchart of patients included in the CRITICS trial. PM: peritoneal metastasis; NEC: neuro-endocrine carcinoma.

Of the 788 patients included and randomized in the CRITICS trial, 636 underwent surgery with curative intent. After the exclusion of patients with neuroendocrine carcinomas (*n* = 5; 1%), or peritoneal metastasis detected during histopathological examination of the resection specimen (*n* = 14; 2%) and post-operative deaths (*n* = 11; 2%), 606 patients were included in this analysis (Figure 1). Baseline characteristics and tumor characteristics are summarized in Table 1 and Table 2, respectively.

### 3.1. Cumulative Incidence of Peritoneal Metastasis

During follow-up, 97 of the 606 patients (16%) had developed peritoneal metastasis as a first event. In 64 of those 97 (66%), the peritoneum was the only site of distant recurrence. Cumulative incidence of metachronous isolated peritoneal metastasis was 9% (95% CI 7–11%) in the first 2 years and 11% (95% CI 8–13%) in the first 5 years after surgery.

### 3.2. Uni- and Multivariable Analysis

A univariable analysis disclosed several factors associated with metachronous isolated peritoneal metastasis (Table 3). Younger age (<60 years), proximal tumor localization, diffuse/mixed histological subtype, ypT4 tumor stage and lymph node involvement (LN^high^) were identified as significant risk factors for metachronous isolated peritoneal metastasis. Sex, allocated treatment arm and the number of received cycles of preoperative chemotherapy, lympathic invasion and vascular invasion were not associated with metachronous isolated peritoneal metastasis.

**Figure 2:**
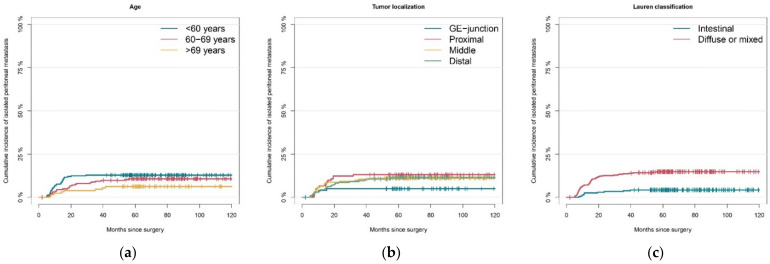

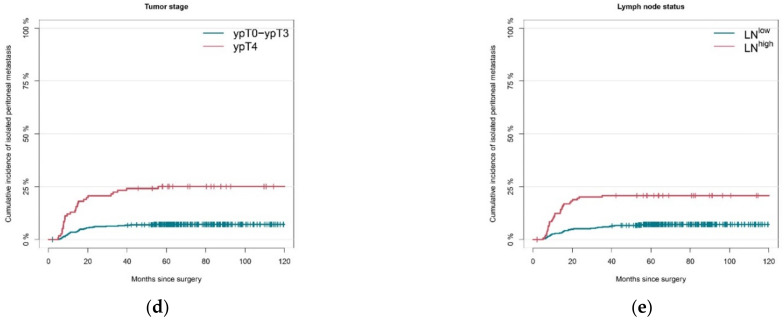
Cumulative incidence plots of metachronous isolated peritoneal metastasis per risk factor. (**a**) Age; (**b**) Tumor localization; (**c**) Histological subtype according to Lauren classification; (**d**) Tumor stage; (**e**) Lymph node stage. GE-junction, gastroesophageal junction. LN^high^, ypN3 lymph node stage and/or a tumor positive lymph node ratio above 20%; LN^low^, ypN0-2 and a tumor positive lymph node ratio below 20%.

Figure 2 shows cumulative incidences of isolated peritoneal metastasis for all five risk factors found significant in univariable analysis. The two-year cumulative incidence of PM was 12% (95% CI 8–16%) in patients younger than 60 years old, versus 8% (95% CI 4–11%) in patients between 60–69 years old, and 4% (95% CI 1–7%) in patients older than 70 years (Figure 2a). Patients with a GE-junction tumor localization had a significantly lower two-year cumulative incidence of 5% (95% CI 1–9%), versus 12% (95% CI 6–18%) in proximal gastric cancer (Figure 2b). The two-year cumulative incidence of PM in diffuse or mixed type gastric cancer was 13% (95% CI 9–16%), compared to 3% (95% CI 1–6%) in intestinal type gastric cancer (Figure 2c). Furthermore, patients with serosal involvement (ypT4) had a significantly higher two-year cumulative incidence than those with ypT0-3 tumors, i.e., 21% (95% CI 13–28%), versus 6% (95% CI 4–8%) (Figure 2d). Comparable results were observed for lymph node involvement, with a two-year cumulative incidence of 20% (95% CI 14–26%) in LN^high^ tumors, compared to 5% (95% CI 3–7%) in LN^low^ tumors (Figure 2e). All five risk factors were further investigated in multivariable analyses.

A multivariable analysis on peritoneal metastasis revealed diffuse/mixed histological subtype, ypT4 tumor stage and LN^high^ nodal involvement as independent risk factors for peritoneal metastasis. Age and tumor localization lost statistical significance in the multivariable analysis (Table 4).

Histological subtype, tumor stage and lymph node involvement were, likewise, investigated in the multivariable analysis on (concurrent) distant metastasis, locoregional recurrence and death, accounting for metachronous isolated peritoneal metastasis as a competing risk. LN^high^ was found to be an independent risk factor for the ‘other events.’ Both diffuse or mixed histological subtype and ypT4 were not significantly associated with ‘other events’ (Table 5).

### 3.3. Cumulative Incidences in Different Subgroups

**Figure 3:**
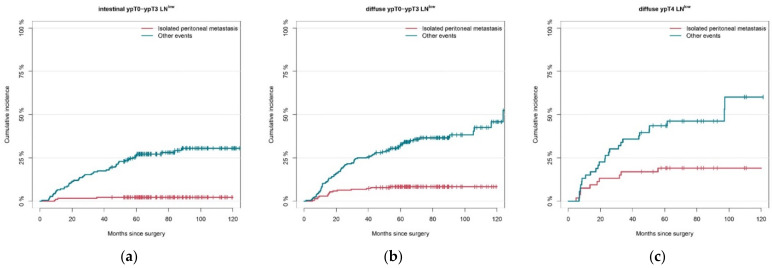

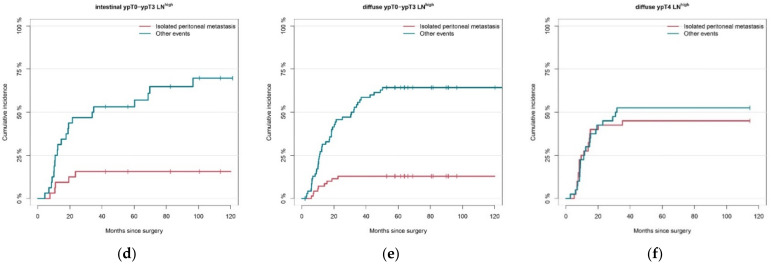
Cumulative incidence plots of metachronous isolated peritoneal metastasis (red) and ‘other events’ (blue) in subgroups with different combinations of independent risk factors for isolated peritoneal metastasis. (**a**) ypT0-ypT3, LN^low^ intestinal GC (*n* = 183); (**b**) ypT0-ypT3, LN^low^ diffuse GC (*n* = 204); (**c**) ypT4, LN^low^ diffuse GC (*n* = 53); (**d**) ypT0-ypT3, LN^high^ intestinal GC (*n* = 32); (**e**) ypT0-ypT3, LN^high^ diffuse GC (*n* = 71); (**f**) ypT4, LN^high^ diffuse GC (*n* = 40).

Figure 3a–f shows the cumulative incidences of both metachronous isolated peritoneal metastasis and ‘other events’ in subgroups of patients that harbored different combinations of the identified independent predictors for peritoneal metastasis. The two subgroups, including patients with LN^low^ or LN^high^ ypT4 intestinal type gastric cancer (*n* = 11 and *n* = 12, respectively), are not shown because of their small sample size.

Isolated peritoneal metastasis was seen least frequently in patients with ypT0-ypT3, LN^low^, intestinal-type tumors with a cumulative incidence of 2% after 2 years (95% CI 0–3%) (Figure 3a). The strongest correlation was seen in patients in whom all risk factors were present with a 43% (95% CI 27–58%) two-year cumulative incidence of peritoneal metastasis (Figure 3f).

As shown in Figure 3a–f, isolated peritoneal metastasis was seen less frequently than ‘other events’ in all investigated subgroups. The cumulative incidence of ‘other events’ was only slightly higher compared to isolated peritoneal metastasis in the ypT4, LN^high^, diffuse type subgroup with two-year cumulative incidences of 45% and 43%, respectively (Figure 3f).

## 4. Discussion

In the last decade, treatment modalities that potentially prevent development of peritoneal metastasis have become available, though at the cost of increased co-morbidity. Therefore, an accurate prediction model to identify patients at a substantial risk for metachronous isolated PM is needed. In this analysis, we evaluated risk factors for the development of metachronous isolated peritoneal metastasis after potentially curative gastric cancer resection in patients treated with preoperative chemotherapy within the CRITICS trial. Diffuse or mixed histological subtype, ypT4 tumor stage and advanced lymph node involvement (ypN3 and/or lymph node ratio above 20%) were identified as independent risk factors for metachronous isolated PM. The combination of these factors was associated with a cumulative risk of 43% of developing metachronous isolated PM.

In our cohort, metachronous PM occurred in 16% of patients after preoperative chemotherapy and potentially curative surgery. The peritoneum was the first and only site of recurrence in 11% of patients. Our findings are consistent with previously reported European studies [4,7]. In accordance with previous results on risk factors for both synchronous and metachronous PM in cohorts of patients not regularly treated with preoperative chemotherapy, we have now found diffuse or mixed type histology (according to Lauren), ypT4 tumor stage and LN^high^ lymph node involvement to be risk factors of metachronous isolated peritoneal metastasis in patients treated with preoperative chemotherapy as well [4,19,22,29].

The effect of age and tumor localization on the occurrence of peritoneal metastasis, as described by previous reports, was not found to be significant in our analyses. Several comprehensive retrospective studies revealed non-cardia gastric cancer and younger age (<60 years) to be significant independent risk factors in their analyses [20,21,22]. These differences may be explained by our smaller cohort compared to these large cancer-registry studies. Furthermore, our study focused exclusively on metachronous peritoneal metastasis as a single site of distant metastasis and, therefore, dissimilarities with previous findings might be found.

The combination of the risk factors found in this study identified a subgroup of patients with a high two-year cumulative incidence of 43% for the development of metachronous isolated PM. Interestingly, advanced lymph node involvement (LN^high^) also seems to be an independent predictor of ‘other events’, such as distant metastases and death. These results broadly support the work of Chang et al., whose single-center study showed advanced N-stage to be an independent risk factor for both peritoneal and distant metastases after D2 surgery in the Asian population [19]. In contrast to our findings, however, the Chang et al. study found that diffuse type histology was a risk factor for both peritoneal and distant metastases. This discrepancy might, on one hand, be explained by differences in tumor biology between Asian and non-Asian populations or, on the other hand, by our focus on peritoneal metastasis in patients treated with preoperative chemotherapy.

Distinct mechanisms of peritoneal spread have been suggested, which match with the risk factors that we found in this study. First, the hypothesis that tumor cells just drop from the serosal surface of the stomach and then outgrow to form peritoneal metastasis, fits with our finding of ypT4 as an important risk factor for PM development [22]. Second, surgical dissection of lymph vessels draining tumors with lymph node involvement may contribute to postoperative peritoneal tumor spread [4]. Moreover, it has been hypothesized that diffuse type gastric cancer has more epithelial-to-mesenchymal transformation characteristics than intestinal type gastric cancer. This may contribute to the successful adherence of diffuse type gastric cancer tumor cells onto the peritoneal surface [30]. This, in combination with the more discohesive growth pattern in diffuse type gastric cancer, might explain the increased risk of metastatic spread compared to the intestinal type. 

Interestingly, the current analysis on risk factors for metachronous isolated peritoneal metastasis of the CRITICS trial did not reveal significant differences between the two allocated treatment arms. Results of the per-protocol analysis of the CRITICS trial, however, suggest that the administration of adjuvant chemotherapy reduces the risk of peritoneal recurrence in general [28]. This might potentially be explained by the fact that we differentiated for the presence or absence of concurrent distant metastases. Moreover, the effect of the adjuvant treatment was not taken into account in our analysis. There is growing evidence that in non-Asian study populations, only 40–50% of gastric cancer patients completes the scheduled adjuvant treatment [2,3,31]. Therefore, future treatment strategies will focus more on neo-adjuvant regimens [32,33].

Within that setting, it might be interesting to investigate the effect of adjuvant intraperitoneal chemotherapy on the risk of metachronous isolated peritoneal metastasis in high-risk populations. Several small studies have already investigated the effect of prophylactic HIPEC in Asian gastric cancer patients and exhibited positive results [16]. However, most studies had a high risk of bias and did not take preoperative chemotherapy into account. A small Italian study showed encouraging results after prophylactic HIPEC in neo-adjuvant treated T3/T4 patients [34]. Likewise, the PERISCOPE-I trial showed promising survival results in neo-adjuvant treated patients with limited peritoneal disease. This is currently being further investigated by the PERISCOPE-II trial [11,15].

The previously described mechanisms of peritoneal spread suggest that microscopic peritoneal disease at the time of surgery can cause metachronous PM in preoperative treated gastric cancer patients. We hypothesize that adjuvant treatment of the peritoneal surface with, for example, intraperitoneal chemotherapy might eradicate these as yet undetectable micrometastasis and, thereby, prevent metachronous PM after preoperative chemotherapy and potentially curative surgery. Since intraperitoneal chemotherapy is associated with considerable morbidity, and our results also suggest that cumulative incidence of ‘other events’ increases simultaneously with cumulative incidence of metachronous isolated peritoneal metastasis in all investigated subgroups, only patients at high risk for metachronous isolated peritoneal metastasis should be considered for this potential adjuvant treatment [16,18]. Patients with tumors positive for all three risk factors had the highest cumulative incidences of metachronous isolated peritoneal metastasis. Therefore, this subgroup of patients might be of interest to additionally treat with prophylactic adjuvant intraperitoneal chemotherapy to prevent metachronous isolated peritoneal metastasis.

Implementation of the identified pathological risk factors in a clinical trial might be challenging, since most factors are not known at the time of surgery and, therefore, not directly applicable as potential inclusion criteria at time of diagnosis. This would mean that re-intervention is needed for the administration of intraperitoneal chemotherapy. One could argue that intraperitoneal chemotherapy would be most effective if administered simultaneously with the gastric cancer resection. However, in the COLOPEC study on adjuvant HIPEC in patients with locally advanced (T4) colon cancer, the incidence of post-operative complications in patients who underwent simultaneous HIPEC was 88%, versus 6% in patients who underwent HIPEC at a second operation [35]. Such a staged approach might, therefore, be the preferred approach for prophylactic intraperitoneal chemotherapy in the gastric cancer population as well.

The currently recruiting French GASTRICHIP trial is a prospective, randomized phase III clinical that aims to evaluate the effect of HIPEC in patients with T4 and/or lymph node positive gastric cancer, with or without positive cytology at peritoneal washing [17]. In this study, HIPEC is performed immediately after gastrectomy. The 7th and 8th versions of the AJCC TNM classification consider tumor positive cytology as a metastatic disease [27,36]. The French trial can, therefore, not be considered a completely prophylactic study. Future studies should distinguish between patients with and without any signs of peritoneal dissemination at time of surgery.

## 5. Conclusions

In conclusion, diffuse or mixed tumor type gastric cancer, ypT4 and advanced lymph node involvement were identified as independent risk factors for the occurrence of metachronous isolated peritoneal metastasis in a large cohort of gastric cancer patients treated with preoperative chemotherapy and potentially curative resection. The combination of these factors might identify a subgroup of gastric cancer patients that could benefit from preventive treatment strategies. Molecular characterization can potentially further improve the risk model of this subgroup. The effect of prophylactic HIPEC in patients with resectable gastric cancer who are positive for all three identified risk factors on the development of peritoneal metastasis, therefore, warrants further investigation.

## Figures and Tables

**Table 1:** Baseline and treatment characteristics of all included patients.

Factor	All Patients (*n* = 606)
Age in years	
Median (IQR)	62 (54–68)
Age in categories	
<60 years	250 (41%)
60–69 years	227 (38%)
≥70 years	129 (21%)
Sex	
Male	407 (67%)
Female	199 (33%)
Country	
Netherlands	473 (78%)
Sweden	118 (20%)
Denmark	15 (2%)
WHO (baseline)	
0	419 (69%)
1	154 (26%)
Unknown	33 (5%)
Allocated postoperative treatment	
Chemoradiotherapy	310 (51%)
Chemotherapy	296 (49%)
Number of preoperative chemotherapy courses	
1	24 (4%)
2	55 (9%)
3	527 (87%)
Tumor localization	
Gastroesophageal junction	102 (17%)
Proximal	122 (20%)
Middle	173 (29%)
Distal	209 (34%)

IQR: interquartile range.

**Table 2:** Pathological characteristics at time of surgery.

Factor	All Patients (*n* = 606)
Lauren classification	
Intestinal	238 (39%)
Diffuse	269 (45%)
Mixed	37 (6%)
Unknown	62 (10%)
Tumor stage *	
ypT0	40 (7%)
ypTis	5 (1%)
ypT1	84 (14%)
ypT2	85 (14%)
ypT3	276 (45%)
ypT4	116 (19%)
Lymph node stage *	
ypN0	302 (50%)
ypN1	102 (17%)
ypN2	108 (18%)
ypN3	94 (15%)
Lymph node ratio	
0–20%	456 (75%)
21–100%	150 (25%)
Lymphatic invasion	
Negative	405 (67%)
Positive	154 (25%)
Unknown	47 (8%)
Vascular invasion	
Negative	474 (78%)
Positive	79 (13%)
Unknown	53 (9%)

* AJCC 8th edition TNM classification.

**Table 3:** Univariable competing risk analysis on metachronous isolated peritoneal metastasis. ‘Other events’ are competing risk.

Factor	Hazard Ratio	95% CI	*p* Value
Age			
<60 years	#	-	
60–69 years	0.8	0.47–1.35	0.4
≥70 years	0.46	0.21–0.99	0.05
Sex			
Male	#	-	
Female	1.54	0.94–2.52	0.09
Tumor localization			
Gastroesophageal junction	#	-	
Proximal	2.74	0.99–7.53	0.05
Middle	2.28	0.85–6.17	0.1
Distal	2.35	0.89–6.21	0.08
Allocated postoperative treatment			
Chemoradiotherapy	#	-	
Chemotherapy	0.8	0.49–1.31	0.37
Number of preoperative chemotherapy courses			
1	#	-	
2	1.39	0.39–5.00	0.61
3	0.8	0.26–2.49	0.7
Lauren classification			
Intestinal	-	-	
Diffuse or mixed	3.72	1.90–7.29	<0.001
Tumor stage *			
ypT0-pT3	#	-	
ypT4	3.91	2.39–6.39	<0.001
Lymph node involvement			
LN^low^	#	-	
LN^high^	3.22	1.97–5.25	<0.001
Lymphatic invasion			
Negative	#	-	
Positive	0.84	0.49–1.44	0.54
Vascular invasion			
Negative	#	-	
Positive	1.01	0.55–1.81	0.98

# Reference; * according to 8th edition AJCC TNM classification; CI, confidence interval; LN^high^, ypN3 lymph node stage and/or a tumor positive lymph node ratio above 20%; LN^low^, ypN0-2 and a tumor positive lymph node ratio below 20%.

**Table 4:** Multivariable competing risk analysis on metachronous isolated PM. ‘Other events’ are competing risk.

Factor	Hazard Ratio	95% CI	*p* Value
Age			
<60 years	#	-	
60–69 years	1.14	0.66–1.99	0.63
≥70 years	0.65	0.30–1.43	0.29
Tumor localization			
Gastroesophageal junction	#	-	
Proximal	2.38	0.84–6.73	0.1
Middle	1.7	0.60–4.83	0.32
Distal	1.81	0.66–4.96	0.25
Lauren classification			
Intestinal	-	-	
Diffuse or mixed	2.72	1.37–5.42	0.004
Tumor stage *			
ypT0-pT3	#	-	
ypT4	2.66	1.59–4.46	<0.001
Lymph node involvement			
LN^low^	#	-	
LN^high^	2.52	1.50–4.25	<0.001

# Reference; * according to 8th edition AJCC TNM classification; CI, confidence interval; LN^high^, ypN3 lymph node stage and/or a tumor positive lymph node ratio above 20%; LN^low^, ypN0-2 and a tumor positive lymph node ratio below 20%.

**Table 5:** Multivariable competing risk analysis on ‘other events.’ Metachronous isolated PM is competing risk.

Factor	Hazard Ratio	95% CI	*p* Value
Lauren classification			
Intestinal	#	-	
Diffuse or mixed	1.05	0.80–1.37	0.73
Tumor stage *			
ypT0-pT3	#	-	
ypT4	1.36	0.98–1.88	0.06
Lymph node involvement			
LN^low^	#	-	
LN^high^	2.36	1.80–3.10	<0.001

# Reference; * according to 8th edition AJCC TNM classification; CI, confidence interval; LN^high^, ypN3 lymph node stage and/or a tumor positive lymph node ratio above 20%; LN^low^, ypN0-2 and a tumor positive lymph node ratio below 20%.

## Data Availability

No new data were created or analyzed in this study. Data sharing is not applicable to this article.
